# Preoperative alpha fetoprotein, total bilirubin, fibrinogen, albumin, and lymphocytes predict postoperative survival in hepatocellular carcinoma

**DOI:** 10.1002/cam4.6030

**Published:** 2023-05-08

**Authors:** Jia Xu, Shu An, Ying Lu, Laisheng Li, Zhi‐Qi Wu, Hua‐Guo Xu

**Affiliations:** ^1^ Department of Laboratory Medicine the First Affiliated Hospital of Nanjing Medical University Nanjing China; ^2^ Branch of National Clinical Research Center for Laboratory Medicine Nanjing Jiangsu China; ^3^ Department of Laboratory Medicine The First Affiliated Hospital of Sun Yat‐Sen University Guangzhou China

**Keywords:** hepatocellular carcinoma, overall survival, prognosis, score model

## Abstract

**Aims:**

Our study focused on exploring the feasible prognostic laboratory parameters of HCC and establishing a score model to estimate individualized overall survival (OS) in HCC after resection.

**Methods:**

Four hundred and sixty‐one patients with HCC who underwent hepatectomy between January 2010 and December 2017 was enrolled in this investigation. Cox proportional hazards model was conducted to analyze the prognostic value of laboratory parameters. The score model construction was based on the forest plot results. Overall survival was evaluated by Kaplan‐Meier method and the log‐rank test. The novel score model was validated in an external validation cohort from a different medical institution.

**Results:**

We identified that alpha fetoprotein (AFP), total bilirubin (TB), fibrinogen (FIB), albumin (ALB), and lymphocyte (LY) were independent prognostic factors. High AFP, TB, FIB (HR > 1, *p* < 0.05), and low ALB, LY (HR < 1, *p* < 0.05) were associated with the survival of HCC. The novel score model of OS based on these five independent prognostic factors achieved high C‐index of 0.773 (95% confidence interval [CI]: 0.738–0.808), which was significantly higher than those of the single five independent factors (0.572–0.738). The score model was validated in the external cohort whose C‐index was 0.7268 (95% CI: 0.6744–0.7792).

**Conclusion:**

The novel score model we established was an easy‐to‐use tool which could enable individualized estimation of OS in patients with HCC who underwent curative hepatectomy.

## 1 INTRODUCTION

Hepatocellular carcinoma (HCC) accounts for 75%–85% cases of primary liver cancer, ranging the third of cancer death worldwide.^1^ Surgical resection has been the first choice of treatment for early‐stage HCC patients.^2^ However, the risk of death following hepatectomy is up to 70% at 5 years despite advances in diagnostic and surgical techniques. In clinical practice, microvascular invasion, tumor and liver function‐related factors are commonly used prognostic indicators.^3^, ^4^, ^5^, ^6^ In addition to these, laboratory parameters also play a vital role in HCC survival.

Alpha‐fetoprotein (AFP) is the most used serum biomarker for HCC, which has been reported having limited prognostic values in the very early‐stage HCC patients.^7^ Therefore, more prognostic serum indicators are urgently needed to supplement the deficiency of AFP to conduct active interventions which can improve survival rates.

Although several studies have sought to explore the prognostic serum indicators in patients with hepatocellular carcinoma,^7^, ^8^, ^9^ few of laboratory parameters were involved, and the results were inconsistent with each other. In addition, it is a worthy task exploring whether the combined use of these serum indicators can improve the accuracy of the postoperative prognosis of HCC. In that context, it will be of vital importance to develop a feasible model for the prognosis in HCC patients using these serum indicators.

Here, we focused on evaluating the prognostic efficiency of various laboratory parameters in HCC and developed a score model that can predict the survival in HCC patients undergoing curative hepatectomy.

## 2 MATERIALS AND METHODS

### 2.1 Patients

From January 2010 to December 2017, 461 HCC confirmed patients who underwent hepatectomy was enrolled in the investigation in The First Affiliated Hospital of Nanjing Medical University. These patient data were used to identify prognostic indicators for the training of proposed model. Basic information of 177 HCC patients from the First Affiliated Hospital of Zhongshan Medical University between January 2016 and April 2019 were available and used for external validation of the scoring model. The selection criteria were as follows: (1) HCC were diagnosed pathologically; (2) age ≥18 years; (3) meet the “Standardization for diagnosis and treatment of primary hepatic carcinoma (2019 edition)” for surgical resection.^10^ Exclusion criteria: (1) other malignant tumors or severe diseases that hinder patient hepatic resection; (2) undergone treatment of HCC‐related before hepatectomy; (3) incomplete clinical data.

The clinical ethics committee of the First Affiliated Hospital of Nanjing Medical University (ethical approval no. 2021‐SR‐253) and the First Affiliated Hospital of Zhongshan Medical University approved all procedures conducted in present study (ethical approval no. IIT‐2022‐538), which conformed to the norm of the Declaration of Helsinki and its later amendments. As a result of retrospective study, the local ethics committee abandoned the requirement of informed consent.

### 2.2 Data collection and follow‐up

The preoperative information of HCC patients were collected, including sex, age, and laboratory examinations including the level of alpha fetoprotein (AFP), carbohydrate antigen 19‐9 (CA19‐9), carcinoembryonic antigen (CEA), aspartate transaminase (AST), alanine transaminase (ALT), lactic dehydrogenase (LDH), γ‐glutamyl transferase (GGT), total bilirubin (TB), direct bilirubin (DB), triglyceride (TG), total cholesterol (TC), albumin (ALB), total protein (TP), creatinine (CR), white blood cell (WBC), lymphocyte (LY), neutrophilic granulocyte (NE), neutrophil‐to‐lymphocyte ratio (NLR), hemoglobin (Hb), red blood cell (RBC) count, red blood cell distribution width (RDW), platelet (PLT) count, Mean platelet volume (MPV), international normalized ratio (INR), prothrombin time (PT), activated partial thromboplastin time (APTT), thrombin time (TT), fibrinogen (FIB), Child‐Pugh grade, clinical stage, BCLC stage, and ECOG score.

The follow‐up was conducted either through telephone or outpatient visits. Overall survival (OS) was defined by the interval between the time of diagnosis and death from any cause.

### 2.3 Statistical analysis

Among patients' characteristics variables, continuous variables were presented as the median and interquartile range. Analysis of variance t‐test was used to compare continuous data. While categorical variables were expressed by counts and percentages which compared using Wilcoxon test. The univariate and multivariate Cox proportional hazards analyses (hazard ratios, HR) were performed to recognize the independent prognostic variable on OS in HCC. Variables with *p* < 0.05 in the univariate analysis were entered into multivariate analysis. For laboratory parameters, the population was divided into three groups (low/middle/high) according to tertiles of these factors. Using the log‐rank test, Kaplan‐Meier survival curves were compared and evaluated survival probabilities. The prediction accuracy of the laboratory markers and the combined scoring model were compared by C‐index. Forest plot was drawn to explore the correlation between different indicators of different concentrations and prognosis of HCC patients. *p* < 0.05 was considered statistically significant. All statistical analyses were carried out using SPSS (26.0 version) and R software (4.2.1 version). The forest plot was drawn by GraphPad Prism8 (8.4.3 version).

## 3 RESULTS

### 3.1 Clinical characteristics of the cohort

A total of 638 subjects (461 of the training cohort and 177 of the validation cohort) with HCC were enrolled in this retrospective study. Baseline characteristics were described in Table 1. In the training cohort, the median age of patients at the time of hepatectomy was 57 and 55 years in the external validation cohort, all range from 48 to 65 years. Five hundred and thirty‐eight (84.33%) patients were male. HBsAg positive was observed in 496 (77.74%) of the patients. Five hundred and seventy‐three patients (89.81%) were Child‐Pugh grade A. Two hundred and ninety‐eight (46.71%) patients were classified as clinical I stage, 143 (22.41%) patients were II stage, and 197 (30.88%) patients were III stage. Fifty‐three (8.31%) patients were in BCLC 0 stage, 338 (52.98%) patients were A stage, 146 (22.88%) patients were B stage, and 101 (15.83%) patients were C stage. Five hundred and seventy‐six (90.28%) patients were scored 0, and 62 (9.72%) patients were scored 1. The imaging and pathological parameters information were presented in the Table 1.

**TABLE 1:** Baseline characteristics of patients with HCC in training cohort and validation cohort.

Variables	Training cohort (*n* = 461)	Validation cohort (*n* = 177)	*p* value
Age, years (median/IQR)	57 (48–65)	55 (48–65)	0.788
Sex (% male)	386 (83.73)	152 (85.88)	0.505
HBsAg (% positive)	359 (79.08)	137 (77.40)	0.621
Anti‐HCV (% positive)	13 (2.82)	6 (3.39)	0.737
Cirrhosis (% yes)	336 (72.89)	138 (77.97)	0.221
MVI (% yes)	143 (31.02)	72 (40.68)	0.021
Laboratory parameters (median/IQR)			
AFP, ng/dL	113.7 (7.88–1210.00)	71.1 (8.53–1881.06)	<0.001
CEA, ng/dL	2.4 (1.57–3.63)	2.6 (1.63–4.07)	0.916
CA199, ng/dL	16.9 (10.00–30.35)	9.12 (4.98–23.24)	0.305
ALT, U/L	34.7 (24.30–52.50)	42.0 (27.00–72.00)	0.003
AST, U/L	38.0 (27.50–55.60)	48.0 (32.00–86.00)	<0.001
GGT, U/L	61.6 (32.00–119.10)	97.0 (50.50–180.00)	0.002
LDH, U/L	198 (172.00–235.00)	245.0 (202.50–330.0)	<0.001
TB, μmol/L	14.7 (11.60–20.00)	18.5 (13.60–28.35)	<0.001
DB, μmol/L	5.3 (3.92–7.40)	4.6 (2.80–8.55)	0.017
TP, g/L	70.7 (66.20–74.70)	66.5 (61.95–71.40)	<0.001
ALB, g/L	41.1 (37.90–43.70)	36.4 (32.26–40.20)	<0.001
CR, μmol/L	69.8 (59.97–79.30)	67.0 (57.00–77.00)	0.410
WBC, (×10^9^/L)	5.1 (4.30–6.45)	7.79 (5.65–10.58)	<0.001
LY, (×10^9^/L)	1.42 (1.11–1.84)	1.10 (0.76–1.62)	<0.001
NE, (×10^9^/L)	3 (2.39–4.05)	5.35 (3.65–8.32)	<0.001
NLR	2.14 (1.55–2.95)	4.60 (2.78–8.78)	<0.001
RBC, (×10^12^/L)	4.59 (4.23–4.92)	4.04 (3.40–4.50)	<0.001
HB, g/L	143 (131.00–153.00)	131 (117.00–144.00)	<0.001
RDW, %	13.4 (12.90–14.10)	14 (13.00–15.00)	0.014
PLT, (×10^9^/L)	140 (102.00–186.00)	167 (103.50–230.50)	<0.001
MPV, fL	10.4 (9.30–11.53)	10.0 (9.10–10.90)	<0.001
PT, s	12.3 (11.80–13.00)	12.5 (11.80–13.90)	0.014
INR	1.07 (1.03–1.14)	1.11 (1.04–1.24)	<0.001
APTT, s	27.9 (25.50–30.70)	30.1 (27.20–35.35)	<0.001
FIB, g/L	2.5 (2.09–3.14)	2.85 (2.11–3.69)	<0.001
TT, s	18.3 (17.50–19.30)	18.1 (17.30–19.00)	0.407
Tumor size (cm)	4.72 (3.00–8.23)	7.10 (3.40–10.70)	<0.001
Tumor number (% single)	368 (79.83)	147 (83.05)	0.355
Child‐Pugh grade, *n* (%)			
A	442 (95.88)	131 (74.01)	<0.001
B + C	19 (4.12)	46 (25.99)	
Clinical stage, *n* (%)			
I stage	253 (54.88)	45 (25.42)	<0.001
II stage	96 (20.82)	47 (26.55)	
≥III stage	112 (24.30)	85 (48.02)	
BCLC stage, *n* (%)			
0	40 (8.68)	13 (7.34)	<0.001
A	301 (65.29)	37 (20.90)	
B	62 (13.45)	84 (47.46)	
C	58 (12.58)	43 (24.29)	
ECOG score, *n* (%)			
0	431 (93.5)	145 (81.92)	<0.001
1	30 (6.51)	32 (18.08)	

Abbreviations: AFP, alpha fetoprotein; ALB, albumin; ALT, alanine transaminase; APTT, activated partial thromboplastin time; AST, aspartate transaminase; BCLC, Barcelona Clinic Liver Cancer; CA199, carbohydrate antigen 19–9; CEA, carcinoembryonic antigen; CR, creatinine; DB, direct bilirubin; ECOG, Eastern Cooperative Oncology Group; FIB, fibrinogen; GGT, γ‐glutamyl transferase; Hb, hemoglobin; INR, international normalized ratio; IQR, interquartile range; LDH, lactic dehydrogenase; LY, lymphocyte; MPV, mean platelet volume; MVI, microvascular invasion; NE, neutrophilic granulocyte; NLR, neutrophil‐to‐lymphocyte ratio; PLT, platelet; PT, prothrombin time; RBC, red blood cell; RDW, red blood cell distribution width; TB, total bilirubin; TP, total protein; TT, thrombin time; WBC, white blood cell.

### 3.2 Predictive factors for HCC survival

On univariate analysis for overall survival, variables with *p* < 0.05 including AFP, CEA, CA199, ALT, AST, GGT, LDH, TB, DB, TC, ALB, LY, RDW, PT, INR, FIB, TT, ALB, LY, TT, and then AFP, TB, ALB, LY, FIB were entered into the multivariate analysis. The results demonstrated that AFP (HR 1.002, 95% CI: 1.001–1.002), TB (HR 1.003, 95% CI: 1.000–1.006), FIB (HR 1.362, 95% CI: 1.183–1.567), and ALB (HR 0.941, 95% CI: 0.911–0.970), LY (HR 0.653, 95% CI: 0.492–0.867) were the independent prognostic factors for OS (Table 2).

**TABLE 2:** Univariate and multivariate analyses of prognostic factors for overall survival.

	Univariate			Multivariate		
	HR	95% CI	*p*‐value	HR	95% CI	*p*‐value
Age	0.995	0.983–1.008	0.995			
Sex (male)	1.132	0.767–1.669	0.532			
HBV	1.068	0.736–1.549	0.731			
HCV	0.667	0.247–1.798	0.423			
AFP	1.002	1.001–1.002	<0.001	1.002	1.001–1.002	<0.001
CEA	1.025	1.009–1.042	0.002			
CA199	1.001	1.000–1.002	0.005			
ALT	1.001	1.000–1.002	0.035			
AST	1.002	1.001–1.003	0.000			
GGT	1.001	1.001–1.002	<0.001			
LDH	1.001	1.001–1.001	<0.001			
TB	1.004	1.001–1.006	0.003	1.003	1.000–1.006	0.024
DB	1.006	1.002–1.009	0.003			
TC	1.167	1.001–1.360	0.049			
TG	0.752	0.541–1.047	0.092			
TP	1.003	0.982–1.026	0.768			
ALB	0.943	0.915–0.971	0.000	0.940	0.911–0.970	0.000
CR	0.993	0.984–1.003	0.172			
WBC	1.019	0.962–1.080	0.517			
LY	0.588	0.444–0.778	0.000	0.653	0.492–0.867	0.003
NE	1.051	0.994–1.111	0.078			
NLR	1.017	0.998–1.037	0.076			
RBC	0.995	0.766–1.293	0.971			
HB	0.996	0.987–1.005	0.360			
RDW	1.115	1.005–1.238	0.040			
PLT	1.002	1.000–1.003	0.106			
MPV	1.044	0.949–1.148	0.381			
PT	1.254	1.110–1.416	0.000			
INR	14.560	3.600–58.890	0.000			
APTT	1.020	0.990–1.051	0.196			
FIB	1.460	1.268–1.681	<0.001	1.362	1.183–1.567	<0.001
TT	0.883	0.795–0.980	0.020			

### 3.3 Optimal values of the independent prognostic factors for OS

The data were divided into three groups of low, middle, and high in tertile, which showed good prognostic classification for HCC patients in this study. AFP was <20, 20–600, and >600 (ng/dL); TB was <12.5, 12.5–17.6, and >17.6 (μmol/L); ALB was <39.0, 39.0–42.8, and >42.8 (g/L); LY was <1.20, 1.20–1.66, and >1.66 (109/L); FIB was <2.22, 2.22–2.86, and >2.86 (g/L).

**FIGURE 1:**
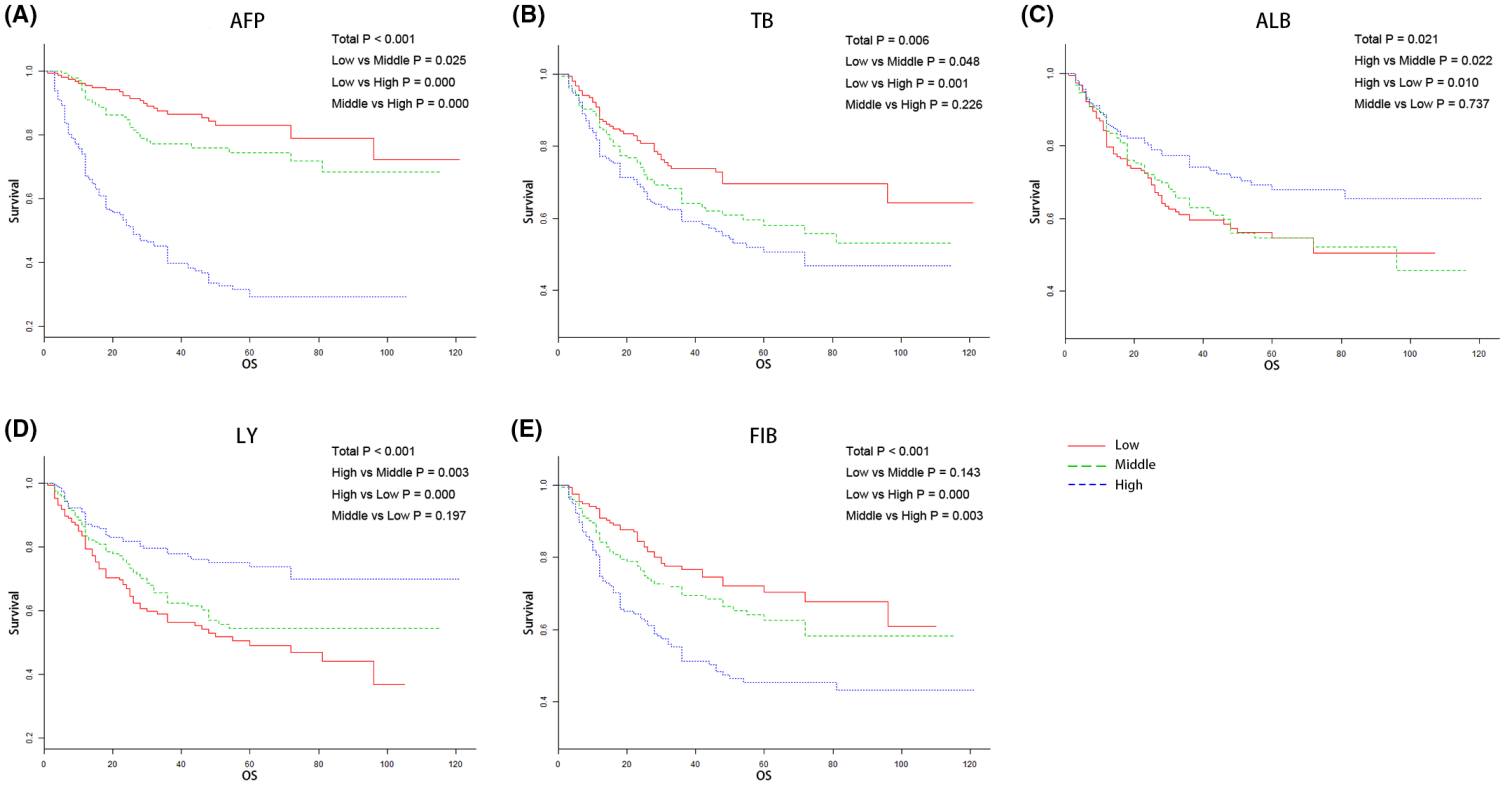
Kaplan‐Meier analysis of HCC patients stratified according to (A) AFP, (B) TB, (C) ALB, (D) LY, (E) FIB.

Patients with lower AFP levels had significantly higher survival probability compared with those higher AFP ones (*p* < 0.001, Figure 1A). The result same as TB and FIB (*p* = 0.006, Figure 1B and *p* < 0.001, Figure 1E). In contrast, the remarkably higher survival probability was ascertained in higher ALB and LY groups (ALB, *p* = 0.021; LY, *p* < 0.001, Figure 1C,D).

### 3.4 Scores of predictive accuracy for OS

**FIGURE 2:**
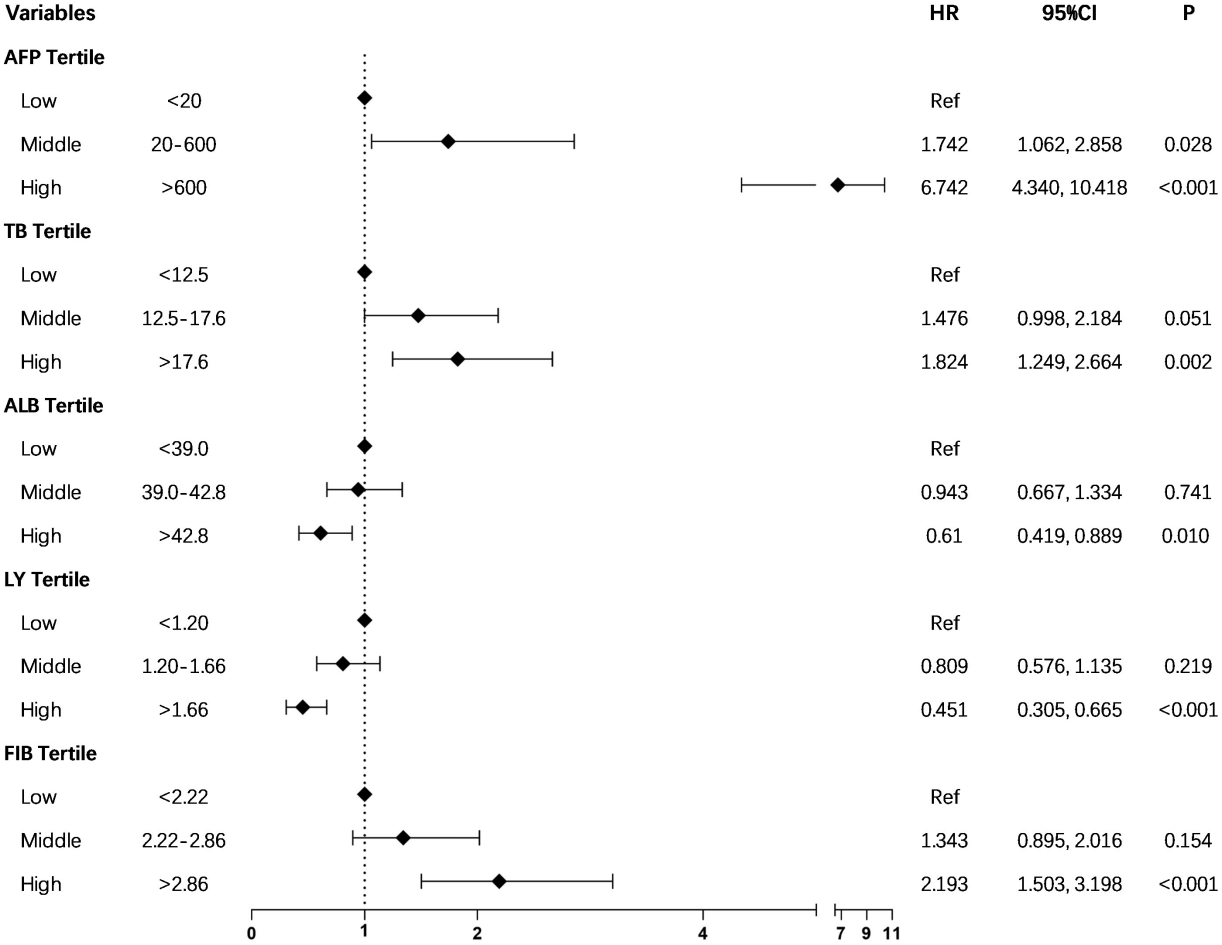
The forest plot for overall survival according to subgroups. Ref, reference.

**FIGURE 3:**
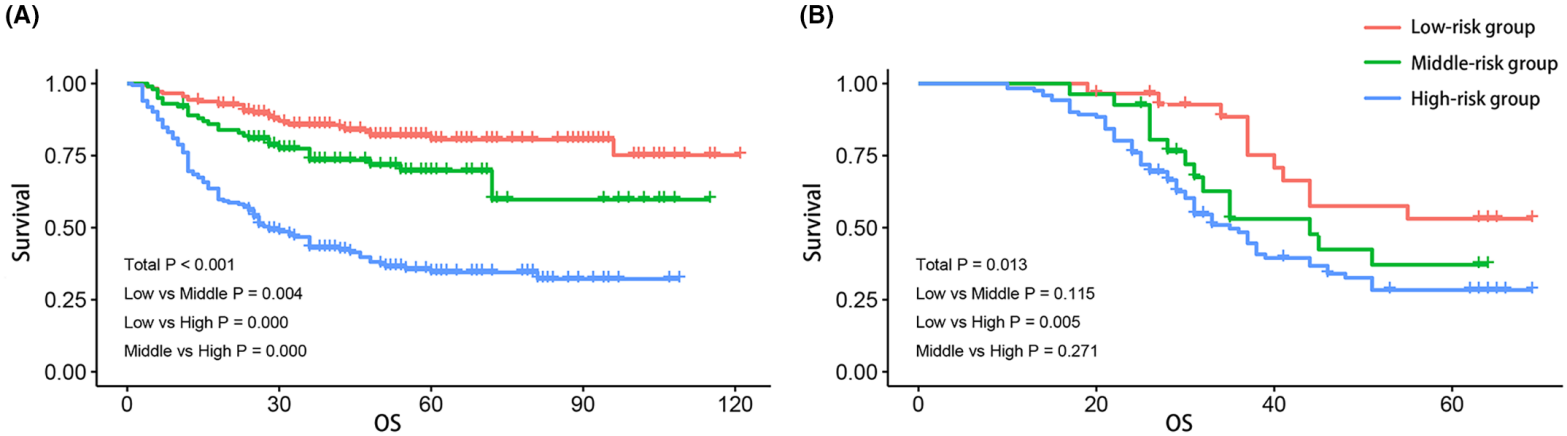
Kaplan‐Meier analysis of HCC patients stratified by the score model. (A) training cohort; (B) external validation.

AFP, TB, ALB, LY, and FIB were used for the score base on the hazard ratios of the significant variables (Table 3). Cox regression analysis of the data from the three groups revealed that HR > 1 for the high group of AFP, TB and FIB, and HR < 1 for the high ALB and LY groups, only the HR of AFP medium group >1, and the HR for other medium groups was across 1, with the low values as reference (Figure 2). With this result the three groups were scored. Therefore, according to scores of the sum of the HCC patient was divided low‐risk group were <10, middle‐risk group were equal 10 and high‐risk group were >10. In the training cohort, there were respectively 182, 99, and 180 patients in the groups defined above (Figure 3A). The median OS of the risk groups was 46, 36, and 26 months, respectively (*p* < 0.001). In the validation cohort, the low‐, middle‐, and high‐risk group included 29, 26, and 122 patients, respectively (Figure 3B). The median OS of the risk groups was 55, 44, and 35 months, respectively (*p* < 0.001).

**TABLE 3:** Models for independent predictive factors.

	Range	Score
AFP (ng/dL)		
Low	<20	1
Middle	20–600	2
High	>600	3
TB (μmol/L)		
Low	<12.5	1
Middle	12.5–17.6	2
High	>17.6	3
ALB (g/L)		
Low	<39.0	3
Middle	39.0–42.8	2
High	>42.8	1
LY (10^9^/L)		
Low	<1.20	3
Middle	1.20–1.66	2
High	>1.66	1
FIB (g/L)		
Low	<2.22	1
Middle	2.22–2.86	2
High	>2.86	3

### 3.5 Comparison of predictive for OS between the scores and AFP

Furthermore, the discrimination of the score model and the five independent risk factors have been compared. In the training cohort, the C‐index of score model was 0.773 (95% CI: 0.738–0.808), which was superior to the C‐index of AFP (0.738, 95% CI: 0.702–0.774), or that of other biomarkers (TB 0.572, ALB 0.583, LY 0.585, FIB 0.620). In the low AFP group, there were 154 patients, 153 patients in the middle AFP group, and 154 patients in the high AFP group. The median OS of the three groups was 44, 42, and 23 months, respectively (*p* < 0.001).

In the validation cohort, the C‐index of score model was 0.727 (95% CI: 0.674–0.779). Sixty‐two patients were in the low AFP group, 58 patients in the middle, and 57 patients in the high. The median OS of the three groups was 40, 38, and 31 months, respectively (*p* < 0.001).

## 4 DISCUSSION

In this study, routine laboratory parameters were used to predict the outcome of HCC. Our finding showed that high AFP, TB, FIB and low ALB, LY were associated with the survival in HCC patients. Furthermore, AFP, TB, ALB, LY, and FIB were considered as independent prognostic factors for the survival of HCC. A better judgment of prognosis by scoring model, it could serve as an easy‐to‐use tool to assist surgeon in preoperative counseling of HCC patients.

Hepatic resection is the ideal method for the treatment of HCC, which benefits not only BCLC‐A patients, but even improve the survival of some BCLC‐B patients.^11^ However, the prognosis of patients who received resection remains poor, and these traditional predictive risk factors such as microvascular invasion, tumor and liver function‐related factors are either too subjective or expensive,^12^ which limit their clinical application. Therefore, the identification of reliable and simple prognostic biomarkers is essential for identifying patients with potentially poor prognosis.

In the current study, cox multivariate analysis showed that five factors were associated with prognosis of HCC patients after hepatectomy. AFP, total bilirubin, albumin, lymphocyte, and fibrinogen were the predictive factors for OS. Among these, total bilirubin, albumin, fibrinogen are commonly used indicators for evaluating liver function. Lymphocyte reflects the degree of inflammation. And AFP is a significant serum biomarker for the diagnosis, prognosis, and efficacy monitoring of HCC.^2^, ^13^, ^14^ All the five included indictors are closely related to postoperative liver function or HCC.

AFP is a kind of tumor marker, which was recommended in the guidelines served as a frequently used diagnostic indicator of HCC. With the progress of research, its prognostic efficacy has been gradually explored. At present, AFP is also a well‐known potential risk factor related to the outcome of HCC patients undergoing hepatectomy.^15^, ^16^ Our study suggested that elevated AFP was inversely correlated with the OS, the same result was also observed in other studies.^8^, ^9^, ^17^, ^18^ Moreover, in the subgroup analysis of our study, we found that AFP is a risk predictor for HCC patients' survival in different concentration ranges. However, although AFP is a widely used laboratory indicator for HCC diagnosis and prognosis in clinical practice, its efficacy remains poor.^19^ Recently, more and more studies showed that the combined model is more effective. The prediction efficiency of the score model established in our study was superior to that of the single AFP, which also proved that the prediction efficiency of the combined model is better than that of the single index model.

Total bilirubin is considered a potentially immunosuppressive toxic substance, which can lead to innate immune abnormalities, cytotoxicity, and abnormal production of pro‐inflammatory cytokines. The elevated concentration of bilirubin is able to induce regulatory T cell function and expansion. While regulatory T cells participate in the downregulation of lymphocyte response and induction of tolerance, leading to immune deficiency.^20^ Additionally, elevated levels of bilirubin may have toxic effects on cells, potentially leading an inflammatory.^20^ It is well known that cirrhosis caused by inflammation is the major factor in the increased risk of HCC,^2^ and immune deficiency will accelerate the progression of tumor.^21^ We demonstrate that high levels of TB are a prognostic risk factor in HCC patients.

Previous studies have shown that lymphocyte plays an important role in the formation and development of tumors.^22^, ^23^ Antitumor immunity makes HCC with inflammatory cell infiltration have a better survival outcome, which is induced by CD8^+^ and CD4^+^ lymphocytes.^24^ And the change of lymphocyte number in blood may reflect the change of lymphocyte infiltration in tumor.^25^ Although some researchers have demonstrated the association between NLR and the poor outcome of HCC patients,^26^, ^27^ in our research, LY was found to be a risk factor for the survival of HCC patients, which is also consistent with some studies.^28^ Moreover, as is known to all, serum albumin can not only reflect the nutritional status of our body, but also predict survival outcome of HCC patients.^29^ In the multivariate analysis of our study, the close connection between lymphocytes and albumin and overall survival was identified. Additionally, only albumin or lymphocyte count in the high level, they have significant relation to the better prognosis of HCC.

The cellular interaction of FIB can participate in tumor metastasis. Tumor cells can interact with endothelial cells, and platelets in the blood circulation, activate FIB of the platelet particles, and release them into the blood to participate in tumor metastasis.^30^ What is more, FIB can block the killer cytotoxicity of thrombin and protect tumor cells from the damage of immune system.^31^, ^32^ In addition, several meta‐analyses have verified that elevated plasma fibrinogen levels represent as a prognostic marker of worse survival and advanced tumor progression.^33^, ^34^ Moreover, the prognostic role of plasma fibrinogen in patients with HCC undergoing hepatectomy has also been investigated.^35^, ^36^, ^37^ In our study, elevated preoperative FIB is adverse prognostic factors for prognosis in HCC patients. What is more, we further evaluated the value of preoperative plasma fibrinogen in subgroups of different concentration, and the results showed that only at high levels can fibrinogen be considered a risk prognostic indicator for the survival of HCC, which indicated the specific application prognostic feature of fibrinogen.

Nevertheless, several studies have reported that total bilirubin and albumin have no significance to the prognosis of HCC patients with hepatectomy,^8^, ^17^, ^38^ which was contrary to our results. We speculate that the possible reasons are as follows. First, in the previous study, varied methods were used to obtain the cutoff value. We divided the population into three groups according to the factors tertiles, while some studies used normal range as cut‐off values. Second, the different characteristics of population composition may account for this disagreement. The main proportion in our study is hepatitis B‐positive patients (77.87%), while Abe et al^38^ is hepatitis C‐positive. This may be account for the difference. Third, the real answer being concealed by some other effectors, which leads to the controversial role of a specific biomarker in HCC.^39^

Based on the five independent prognostic factors, a score model from a prognostic cohort undergoing hepatectomy was developed. The five indictors can be readily ascertained in clinical practice, making the model feasible in estimating individual risk of OS after hepatectomy. This prognostic score model, which was verified by C‐index, had good prediction accuracy. Furthermore, the comparison of C‐index between the model and each marker (AFP, TB, ALB, LY, and FIB) showed that the performance of the model predicting survival was outperformed to the single marker. Also, despite the statistically significant differences in most demographics (such as tumor size, Child‐Paugh grade, Clinical stage, MVI, and some laboratory indicators) between the external validation cohort and the training cohort, the C‐index of the external cohort was 0.727, which revealed an excellent extrapolation ability of our model. Of note, in the validation cohort, due to the low‐risk group had a survival rate of more than 50% (18/29), we used the mean OS instead of the median OS. What is more, though several studies have developed prognostic model in HCC patients undergoing hepatectomy,^40^, ^41^ they involved imaging indicators, while our model only used laboratory parameters and had better prognostic efficacy.

We are aware of the limitations of this study. First, our conclusion was based on patients with HCC in the dominant region of hepatitis B virus. Further validation will be needed to assess whether this conclusion can be used for other etiologies. Second, as a retrospective study, patient selection bias is inevitable. Also, we were not able to include some unconventional indicators. Third, the external validation cohort requires cases from other centers to identify the model so that can prove the clinical utility of the model.

In conclusion, we have established a laboratory based practical model from a large population‐based cohort to prognosticate HCC patients undergoing hepatectomy. Our novel and straightforward risk classification is superior to currently preoperative prognostic tools. By incorporating 5 common clinical data, the easy‐to‐use tool that estimates individual survival could function as a meaningful tool for surgeons in preoperative counseling.

## AUTHOR CONTRIBUTIONS

**Jia Xu:** Conceptualization (equal); formal analysis (equal); investigation (equal); methodology (equal); writing – original draft (equal). **Shu An:** Conceptualization (equal); formal analysis (equal); investigation (equal); methodology (equal). **Ying Lu:** Conceptualization (equal); formal analysis (equal); investigation (equal); methodology (equal). **Laisheng Li:** Conceptualization (equal); formal analysis (equal); investigation (equal); supervision (equal); validation (equal); writing – review and editing (equal). **Zhi‐Qi Wu:** Conceptualization (equal); formal analysis (equal); investigation (equal); methodology (equal); supervision (equal); writing – review and editing (equal). **Hua‐Guo Xu:** Conceptualization (equal); formal analysis (equal); funding acquisition (equal); project administration (equal); resources (lead); supervision (lead); writing – review and editing (lead).

## FUNDING INFORMATION

This study was supported by the Natural Science Foundation of Jiangsu Province of China (BK20181492), Jiangsu Provincial Research Hospital (YJXYY202201) and Jiangsu Provincial Medical Key Discipline (Laboratory) (ZDXK202239).

## CONFLICT OF INTEREST STATEMENT

The authors have no conflict of interest to declare.

## ETHICAL STATEMENT

Approval of the research protocol: The clinical ethics committee of the First Affiliated Hospital of Nanjing Medical University (Ethical approval No.2021‐SR‐253) and the First Affiliated Hospital of Zhongshan Medical University approved all procedures conducted in present study (Ethical approval No.IIT‐2022‐538), which conformed to the norm of the Declaration of Helsinki and its later amendments.

## INFORMED CONSENT

For this retrospective study, the requirement for informed consent was waived by the local ethics committee.

## Data Availability

The data that support the findings of this study are available on request from the corresponding author. The data are not publicly available due to privacy or ethical restrictions.
